# BRIDGE GERIATRIC WORKFORCE EDUCATION CURRICULUM BETWEEN 4M COMPETENCIES AND INTERDISCIPLINARY EDUCATION

**DOI:** 10.1093/geroni/igad104.1434

**Published:** 2023-12-21

**Authors:** Ji Yoo, Jay Shen, Ian choe, maryam Tabrizi, Iulia Ioanitoaia-Chaudhry

**Affiliations:** Kirk Kerkorian School of Medicine at UNLV, Las Vegas, Nevada, United States; School of Public Health, UNLV, Las Vegas, Nevada, United States; Optum Nevada, Las Vegas, Nevada, United States; University of Nevada in Las Vegas, Las Vegas, Nevada, United States; VA Southern Nevada Healthcare System, North Las Vegas, Nevada, United States

## Abstract

In a provider shortage area, education priority strategy is more practical rather than structure investment priority strategy. In aims of establishing Age-Friendly Community in this state where primary care provider number per capita ranks 50th state, 4Ms (What Matters, Mobility, Medication, Mentation) based interdisciplinary education across medicine, dental, pharmacy, nursing, social work, physical therapy were developed and implemented. We evaluated the impact of 4Ms application on healthcare providers education by measuring served patient outcomes (1) CMS defined Merit Incentive Payment System, patient satisfaction, and healthcare cost and (2) education outcomes of healthcare providers using 4M education competencies. We adopted STEPPS interprofessional communication skill toolkit and applied simulation training with standardized patients.

